# Metabolomics‐driven approaches for identifying therapeutic targets in drug discovery

**DOI:** 10.1002/mco2.792

**Published:** 2024-11-11

**Authors:** Shanshan Pan, Luan Yin, Jie Liu, Jie Tong, Zichuan Wang, Jiahui Zhao, Xuesong Liu, Yong Chen, Jing Miao, Yuan Zhou, Su Zeng, Tengfei Xu

**Affiliations:** ^1^ Research Center for Clinical Pharmacy College of Pharmaceutical Sciences Zhejiang University Hangzhou Zhejiang China; ^2^ College of Pharmaceutical Sciences Zhejiang University Hangzhou Zhejiang China; ^3^ Department of Radiology and Biomedical Imaging PET Center Yale School of Medicine New Haven Connecticut USA; ^4^ School of Basic Medical Sciences Zhejiang Chinese Medical University Hangzhou China; ^5^ Cangnan County Qiushi Innovation Research Institute of Traditional Chinese Medicine Wenzhou Zhejiang China

**Keywords:** artificial intelligence, drug development, metabolomics, single‐cell metabolomics, target identification

## Abstract

Identification of therapeutic targets can directly elucidate the mechanism and effect of drug therapy, which is a central step in drug development. The disconnect between protein targets and phenotypes under complex mechanisms hampers comprehensive target understanding. Metabolomics, as a systems biology tool that captures phenotypic changes induced by exogenous compounds, has emerged as a valuable approach for target identification. A comprehensive overview was provided in this review to illustrate the principles and advantages of metabolomics, delving into the application of metabolomics in target identification. This review outlines various metabolomics‐based methods, such as dose–response metabolomics, stable isotope‐resolved metabolomics, and multiomics, which identify key enzymes and metabolic pathways affected by exogenous substances through dose‐dependent metabolite–drug interactions. Emerging techniques, including single‐cell metabolomics, artificial intelligence, and mass spectrometry imaging, are also explored for their potential to enhance target discovery. The review emphasizes metabolomics' critical role in advancing our understanding of disease mechanisms and accelerating targeted drug development, while acknowledging current challenges in the field.

## Graphical abstract

Various metabolomics‐based methods were outlined that have been employed for target identification, such as dose–response metabolomics, stable isotope‐resolved metabolomics, mass spectrometry imaging metabolomics. Furthermore, the emerging potentials of target identification based on metabolomics, such as artificial intelligence, single‐cell metabolomics, and so on, have effectively enhanced the target identification process. Metabolomics has become a powerful tool in drug research and development.

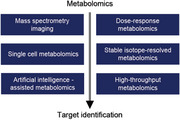

## 1 INTRODUCTION

Drug targets are critical to drug development, representing molecular sites where drugs interact with the body. These targets typically include key proteins, enzymes, or cellular components involved in disease progression.^1^ Early‐stage drug development focuses on identifying such targets to design novel therapeutics. By 2022, the therapeutic target database cataloged 498 targets, with 2797 newly approved drugs acting on these sites.^2^ For example, ivosidenib targets mutated IDH‐1/2 enzymes to treat acute myeloid leukemia (AML),^3^ while various drugs target the glucagon receptor (e.g., glucagon intranasal, rDNA‐origin glucagon).^4^

In addition to targeting known sites, discovering more efficient targets is key to understanding mechanisms of action (MoAs) for exogenous compounds, a long‐standing focus in pharmacology. Recent advances, such as chemical proteomics, have successfully elucidated drug targets and MoAs, including FKBP12 for FK506 and rapamycin,^5^, ^6^ and eIF3i for lenalidomide via a lenalidomide probe approach.^7^ Virtual screening or in silico methods have successfully identified protein targets for numerous compounds, such as the interaction between MEK1, EGFR, and Aurora A with the anticancer treatment, ginsenosides.^8^ Similarly, B‐cell lymphoma 2 and related proteins were identified as targets for porphyrin photosensitizers.^9^ Systems biology approaches using transcriptomics and proteomics have identified potential therapeutic targets, such as ANXA1 and CLEC3B for COVID‐19.^10^ These advancements have significantly enriched and expedited the drug discovery process.

However, protein‐level focus in these methods may overlook phenotypic changes caused by exogenous compounds, increasing the risk of false positives. Phenotypic drug discovery offers an alternative, with methods like high‐content analysis,^11^, ^12^, ^13^ and probe hybridization‐based drug screening by sequencing (PHDs‐seq),^14^ enabling novel drug discoveries. An analysis of United States Food and Drug Administration approved drugs from 1999 to 2008 reveals that target‐based screening approaches have only discovered 17 first‐in‐class drugs. This may be attributed to a focus on “me‐too” drugs. Conversely, phenotype‐based screening has led to the development of 28 first‐in‐class drugs.^15^ Metabolomics, closely tied to phenotypic changes, has emerged as a vital tool in systems biology. It is often combined with other omics techniques, like proteomics and transcriptomics, as part of a multiomics study. With its origins dating back to the 1970s, metabolic profiling can be attributed to the inception of metabolomics,^16^, ^17^ with the advantages of universality, meeting the needs of large samples and rapid screening. Therefore, metabolomics is finding to be used in drug development,^18^ precision medicine,^19^, ^20^ agronomy,^21^ food science,^22^ and other fields.

Recent applications of metabolomics for small molecule target identification have shown advantages in high‐throughput, rapid, and sensitive analyses.^23^, ^24^ Though metabolomics’ role in disease mechanisms and drug discovery has been discussed,^19^, ^25^, ^26^, ^27^ a systematic review of emerging metabolomics‐based target identification methods is lacking. This review provides an overview of these methods and explores technologies such as mass spectrometry imaging (MSI), artificial intelligence (AI), and single‐cell analysis in metabolomics‐based target identification.

## 2 DRUGGABLE TARGETS OF SMALL MOLECULES

A drug target is closely linked to a disease process and serves as the main site of action for drug compounds. Modulation of its activity or function can produce therapeutic benefits. Effective drug targets should exhibit properties such as efficacy, safety, and novelty.^28^ Based on their MoAs, drug targets can include enzymes, substrates, metabolites, receptors, ion channels, transport proteins, RNA, DNA, ribosomes, and monoclonal antibody targets. Key protein classes in drug target discovery include proteases, kinases, G protein‐coupled receptors, and nuclear hormone receptors.^29^

Target‐based drug discovery focuses on developing therapies by regulating specific molecular mechanisms, allowing drugs to correct disrupted metabolic and signaling pathways involved in diseases. Enzymes, as primary catalysts in metabolic processes, play a central role in maintaining cellular energy balance and regulating these pathways.^30^, ^31^ Certain enzymes are also directly implicated in disease initiation by producing pathological mediators or regulatory factors. However, identifying suitable drug targets remains a challenge due to a lack of sufficient experimental validation.^32^

The complexity of drug target–disease relationships arises from the involvement of multiple molecular pathways and targets in most diseases.^33^ For instance, Alzheimer's disease (AD) is characterized by a complex pathophysiology, including disrupted neurotransmission and increased oxidative stress, which has led to the development of multitarget drug strategies.^34^ Advances in omics technologies have revolutionized the ability to screen biological samples at multiple levels, including genomic, transcriptomic, proteomic, and metabolomic analyses, enabling a deeper understanding of biological targets and their interactions across these networks.^35^

## 3 OVERVIEW OF METABOLOMICS

**FIGURE 1:**
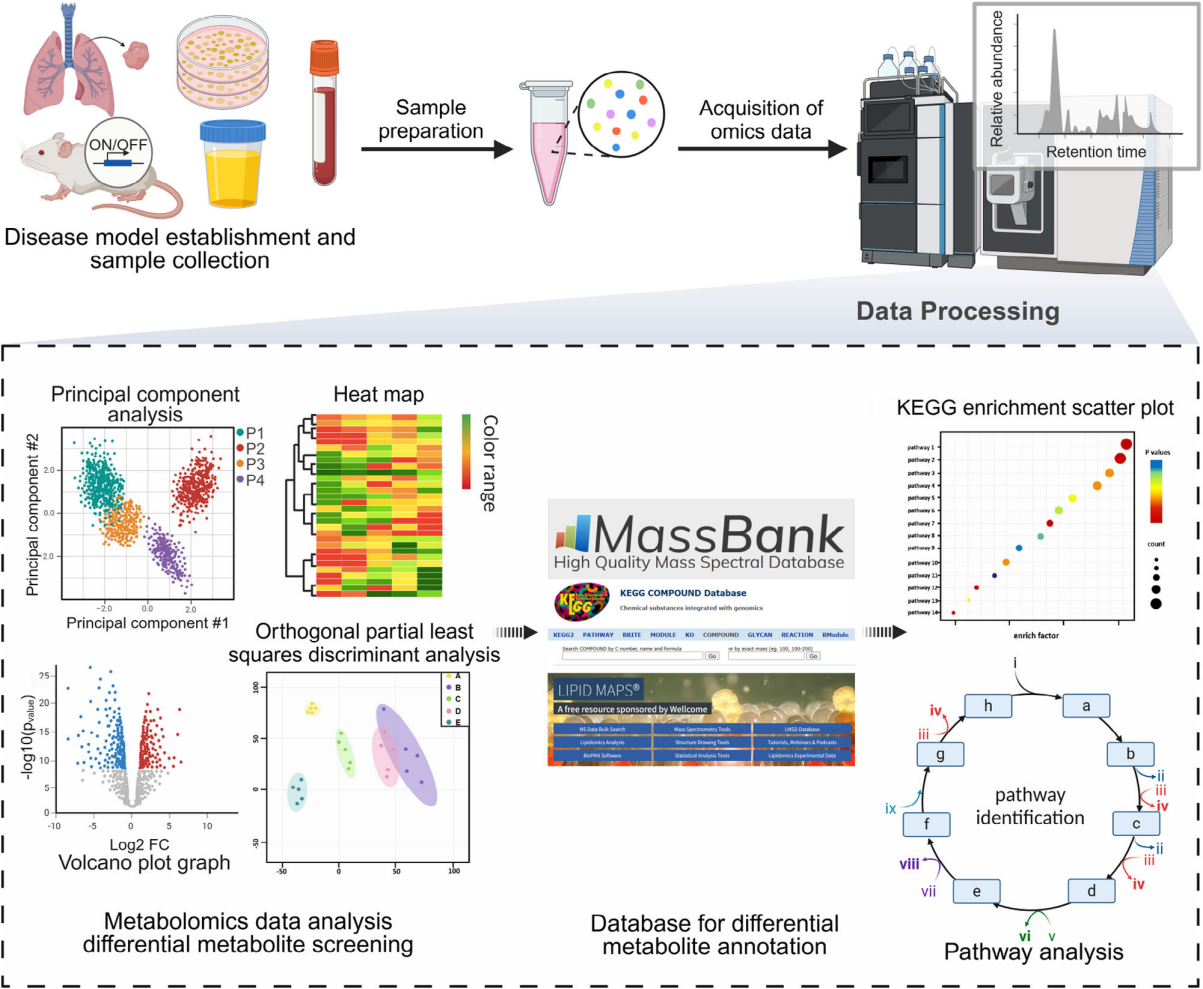
General workflow for metabolomics analysis of biological samples. It divides into four parts: (1) sample collection and preparation; (2) metabolic profiling; (3) data processing and analysis; and (4) pathway analysis (created by Biorender; https://biorender.com).

Metabolomics is a valuable method for studying small‐molecule metabolites (<1000 Da), helping to identify biomarkers and uncover biological processes^36^ (Figure 1), while its broad analysis across various samples is essential for understanding metabolic and physiological shifts.^37^

Metabolomics can be divided into lipidomics and hydrophilic metabolomics, depending on metabolite properties.^38^, ^39^ Given the complexity of the organism's lipidome, lipidomics has evolved into a distinct discipline. Also, approaches are categorized into targeted and untargeted metabolomics based on the research objectives. Targeted metabolomics focuses on specific molecules, offering detailed insights into metabolic pathways. While untargeted metabolomics provides broad screening, detecting thousands of metabolites simultaneously to discover novel dysregulations.^40^, ^41^ Recently, pseudotargeted metabolomics, which integrates both approaches, enables high coverage and accurate quantification, making it ideal for large‐scale studies.^42^

### 3.1 Preparation and technology platforms for metabolomics

Metabolites, as products of the genome and transcriptome, are closely tied to phenotypes, reflecting an organism's health and disease state. Their quick response to stimuli, like drug treatments, makes them ideal for tracking drug effects, while metabolomics helps decode complex biological systems by mapping metabolic pathways.

Metabolomics can detect various types of sample, including blood (serum and plasma), urine,^43^, ^44^ cerebrospinal fluid,^45^, ^46^ lymph, bile, feces,^47^ saliva,^48^, ^49^ cells,^50^, ^51^, ^52^, ^53^ and tissues.^54^, ^55^ Metabolites are prone to degradation due to environment (e.g., sample preparation, storage, and analytical conditions), leading to inconsistent results. Proper sample preparation is crucial for experiment success, as it concentrates target metabolites, removes interferences, and ensures comprehensive metabolite coverage for analysis. Common methods include stabilizing samples through low temperatures, freeze‐drying, or buffering, followed by extraction and analysis, with additional steps like filtration, centrifugation, fractionation, preconcentration, or derivatization as needed.^56^ Commonly used methods for extracting metabolites include organic solvent‐based deproteinization, liquid–liquid extraction,^57^ solid‐phase extraction,^58^, ^59^ and others. Additionally, innovative techniques like solid‐phase microextraction (SPME),^60^ microwave‐assisted extraction,^61^, ^62^ headspace SPME,^60^, ^63^ direct immersion SPME,^60^ ultrasound‐assisted extraction,^56^, ^64^ and enzyme‐assisted extraction^56^ have been attempted to be applied to sample preparation for metabolomics.^65^

Automation in metabolomics has advanced analytical platforms, improving reliability, reproducibility, and throughput.^66^ Mass spectrometry (MS) and nuclear magnetic resonance (NMR) are the primary detection technologies, while liquid chromatography (LC), gas chromatography (GC), and capillary electrophoresis (CE) are commonly used for metabolite separation.^67^, ^68^

MS‐based metabolomics has become the leading platform due to its exceptional sensitivity, selectivity, and wide dynamic range, utilized either directly (direct infusion [DI] MS) or in conjunction with chromatographic techniques.^67^, ^68^ Initially, GC–MS was favored for its high selectivity, repeatability, and structured databases,^69^ but LC–MS has since become the most widely used method due to its ability to handle nonvolatile compounds. LC–MS has the advantage of efficient separation and detection capabilities for a wide range of molecules.^70^, ^71^ CE–MS is particularly suited for analyzing polar/ionic metabolites,^72^ though its use is limited by technical challenges and lower reproducibility,^73^ NMR remains valuable for absolute quantification^75^ and de novo structure elucidation,^26^ complementing MS by detecting nonionizable compounds.^76^ Other emerging technologies include Fourier transform infrared spectroscopy, MSI, and magnetic resonance imaging, which are increasingly integrated into metabolomics workflows.^77^ Advancements in detection sensitivity now allow for the identification of low‐abundance metabolites, facilitating research in drug metabolism and pharmacodynamics, while the increasing focus on metabolic dynamics—affected by factors such as time, dosage, and individual variability—has created a demand for high‐throughput methodologies, spurring innovation in technological platforms.

### 3.2 Data processing and analysis in metabolomics

In metabolomics research, statistical and bioinformatics tools are employed to compare metabolic profiles across different groups, facilitating the identification of significant metabolites, or biomarkers.^77^ Prior to data analysis, raw data preprocessing is essential, involving steps to remove technical variations, errors, normalization, and filtering of irrelevant data.^78^ Normalization is particularly important due to large sample sizes and variations in chromatographic peak areas, with methods such as quality control samples and internal‐standard normalization being utilized for effective data adjustment.^79^

The identification of complex metabolites commonly involves comparing experimental exact masses and MS/MS spectra with data from spectral databases,^80^ including METLIN,^81^, ^82^ NIST, MassBank, HMDB, ReSpect,^83^ MSforID,^84^, ^85^ mzCloud,^84^ and LipidBlast.^85^ Spectral databases and matching algorithms are vital for metabolite identification, supported by software tools for automated data processing and annotation. Widely used tools include XCMS^86^, ^87^, ^88^ and MZmine^89^ for data processing, while Asari,^90^ MetaboAnalyst,^91^, ^92^ MAVEN,^93^ and MS‐DIAL^94^ are also employed for further analysis.

After data acquisition, multivariate data analysis is essential for identifying differential metabolites, employing key methods such as principal component analysis (PCA), partial least squares‐discriminant analysis, orthogonal partial least squares‐discriminant analysis, volcano map, clustering analysis, trend clustering analysis, correlation analysis, and coexpression networks.^95^, ^96^ Identified differential metabolites can serve as potential biomarkers^77^, ^97^ for disease diagnosis, staging, monitoring disease progression, assessing clinical outcomes, and conducting safety evaluations.^98^, ^99^, ^100^, ^101^, ^102^

Metabolic pathway analysis, combined with gene expression data, identifies key enzymes and pathways that link metabolite changes to specific biological processes, thereby elucidating causal relationships and guiding further research. Bioinformatics methods, such as pathway enrichment tests^103^, ^104^, ^105^ and TrackSM,^106^ enhance the analysis of untargeted metabolomics data by accurately associating compounds with metabolic pathways. Key databases, including KEGG (http://www.genome.jp/kegg/),^107^, ^108^, ^117^ Reactome (https://reactome.org),^109^, ^110^, ^111^, ^112^ and BioCyc (https://BioCyc.org),^108^ along with resources like EHMN (http://www.ehmn.bioinformatics.ed.ac.uk) and wikiPathway (https://www.wikipathways.org/), are essential for researchers in exploring metabolic pathways and their biological significance. However, due to the sensitivity of metabolic processes, individual variability in drug metabolism can introduce biases in analytical results. Furthermore, some drugs induce metabolic alterations through target genes or by disrupting membrane structures, rather than through metabolic enzymes, complicating analysis and mechanistic interpretation. This complexity has prompted the emergence of integrative approaches that combine metabolomics with other omics disciplines.

## 4 METABOLOMICS DRIVEN TARGETS IDENTIFICATION

Functional metabolomics, also known as activity metabolomics,^113^ is a specialized discipline that intersects with phenotyping. It focusing on the identification of active metabolites that modulate cellular activities and influence phenotypic outcomes.^114^ This field emphasizes the correlation between metabolic responses and gene regulation to pinpoint key metabolites, referred to as active metabolites, which play crucial roles in disease progression.

Unlike general metabolomics, which catalogues a wide range of differential metabolites, functional metabolomics targets these key active metabolites, allowing for modulation of critical metabolic processes through their supplementation or depletion. This targeted approach can significantly impact disease progression,^120^, ^122^, ^123^, ^124^, ^125^ as demonstrated by Lanzavecchia et al.,^115^ who found that L‐arginine enhances T cell metabolism and antitumor activity. Additionally, Peng et al.^116^ reported that exogenous alanine and glucose, in combination with kanamycin, effectively eliminate antibiotic‐resistant bacteria. Beyond cancer, functional metabolomics can regulate various processes, including intestinal barrier function^117^ and immunometabolism.^115^

**FIGURE 2:**
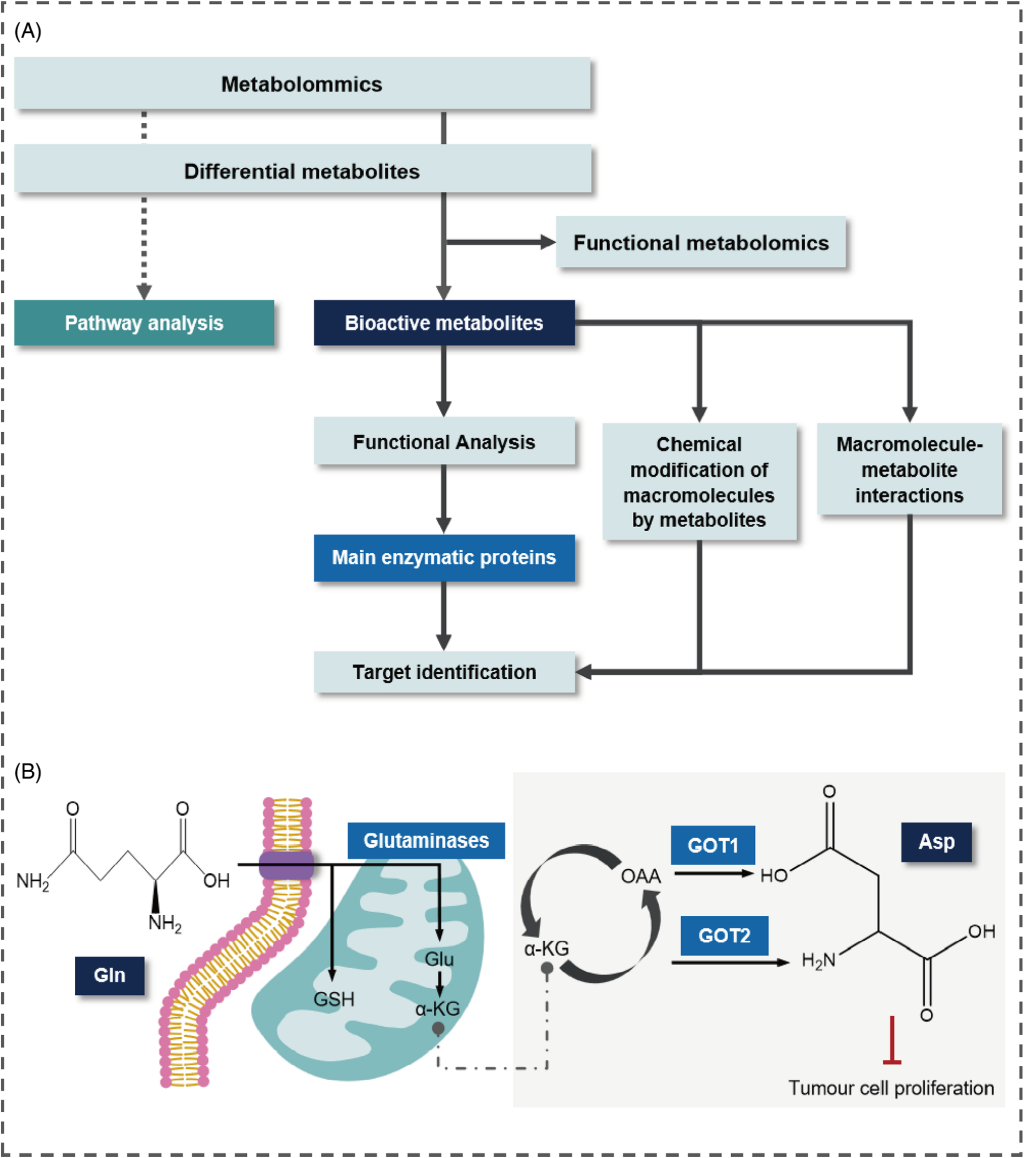
Functional metabolomics for identifying active metabolites and their regulatory roles. (A) Differences between functional and general metabolomics. (B) (1) Glutamine (Gln) levels impact cellular metabolism by influencing the synthesis of key metabolites like glutamic acid (Glu) and α‐ketoglutarate (α‐KG). These changes affect neurotransmission, energy production, and amino acid metabolism, leading to phenotypic alterations in cells. Meanwhile, increased Gln levels stimulate GSH synthesis, aiding in maintaining redox balance and reducing oxidative stress by synthesizing NADPH; (2) aspartate (Asp) level is regulated by proteins GOT1 and GOT2, which inhibit the proliferation of cancer cells.

Numerous active metabolites have been extensively studied. Aspartate serves as an endogenous metabolite that restricts cancer growth,^118^, ^119^ with studies indicating its association with hypoxia‐inducible factor‐1a hinders tumor cell proliferation.^120^ Similarly, glutamine (Gln) has been recognized as a critical regulator of cell growth^121^ and proliferation,^122^ underscoring the functional roles of active metabolites in phenotype modulation (Figure 2B).

Functional metabolomics is essential for identifying potential therapeutic targets by examining the roles of bioactive metabolites in biological processes and their connections to metabolic pathways. By uncovering metabolic vulnerabilities and elucidating key pathways and enzymes, it enables the development of targeted drugs that influence phenotypes, serving as a vital link between phenotypic data and the underlying biological mechanisms associated with metabolic dysregulation^25^ (Figure 2A).

Functional metabolomics has proven instrumental in revealing differences among conditions, such as distinguishing between chronic hepatitis and acute hepatitis in mice^123^ and highlighting gender‐specific variations in amino acids and fatty acid metabolism.^124^ Additionally, it has been applied in agronomy to explore evolutionary differences between species.^125^ Recently, this approach has gained traction as a valuable tool for identifying therapeutic targets, showing great promise for drug discovery; the following sections will discuss the methodologies and concepts relevant to drug target discovery through metabolomics.

### 4.1 Metabolomics facilitates to the discovery of disease targets

Disease targets are essential in preliminary drug discovery research and are pivotal for developing targeted therapies. The distinction between disease and drug targets is crucial given advancements in pharmacological research, particularly in the realms of traditional Chinese medicine (TCM), multitarget discovery, and drug repositioning. Extensive research has unveiled numerous disease‐associated factors and even pathogenic factors that can potentially serve as disease targets.^126^ When these variation factors can be normalized through interactions with drugs, they have druggability and can be further used in drug development. Systematic investigation of disease mechanisms enables precision medicine by identifying optimal targets. Genomics, transcriptomics, and proteomics have been widely used to explore disease pathways and targets,^127^ such as allelic mutations in human leukocyte antigen molecules^128^ and ABCA7 in AD.^129^ However, changes in gene‐protein expression revealed by histological analyses need further experimental validation to establish causality.^126^ Similarly, the redundancy and complexity of biological pathways make it challenging to identify therapeutic targets from gene expression data.

Recently, metabolomics has become an essential field that emphasizes phenotype‐oriented analysis to reveal metabolic changes linked to various diseases. It establishes a correlation between genetic alterations and disease phenotypes, facilitating biomarker discovery and the exploration of disease mechanisms.^127^ Metabolomics investigates the differences in metabolic profiles between healthy and diseased states to identify key metabolites related to diseases, effectively accounting for external influences and individual variability, and enabling the exploration of potential target proteins through pathway association analysis.^130^, ^131^

For instance, TWIK‐related acid‐sensitive potassium channel 1 (TASK‐1 or KCNK3) has been identified as a potential therapeutic target in lung adenocarcinoma, as it inhibits proliferation and glucose metabolism through the activation of the AMPK–TXNIP pathway.^132^ In Kawasaki disease (KD), Wu et al.^133^ found significant pathway differences in patients with coronary artery lesions (CALs) compared with those without, with alterations in phenylalanine metabolism and unsaturated fatty acid biosynthesis. Notably, palmitic acid emerged as the most significantly differentially expressed metabolite, accelerating endothelial cell senescence through reactive oxygen species (ROS) production, thereby presenting a new target for CALs in KD patients.^132^ Additionally, Zheng et al.^134^ observed significantly elevated plasma succinate concentrations in patients with aortic aneurysm and dissection. Analysis of untargeted metabolomics emphasized that succinate as both a biomarker and potential therapeutic target due to its regulatory role via the p38α–CREB–OGDH axis in macrophages, which induces inflammatory changes.^134^ Furthermore, Lu et al.^135^ identified the farnesoid X receptor as a therapeutic target for pancreatic necrosis by modulating bile acid metabolism. Table 1 presents examples of metabolic enzyme targets with therapeutic potential, some of which have been leveraged for drug development.

**TABLE 1:** Selected metabolic enzyme targets with therapeutic potential.

Target ID	Key metabolic changes	Function	Description	Metabolomics method/role
FAAH	Lipid metabolism: The NATs were significantly elevated in the FAAH (−/−) mice^136^, ^137^	Inactivation of FAAH leads to elevated endogenous levels of fatty acid amides, which activate TRP ion channels.^144^, ^145^	FAAH is a potential therapeutic target for a range of nervous system and peripheral disorders.	Characterize the FAAH−NATs system via untargeted metabolite analysis and targeted metabolite analysis performed by isotope‐dilution MS^136^, ^137^
HIF1α	Energy metabolism: Increased NADH/NAD ratio indicates an enhancement of glycolysis and a reduction in the oxidation of glucose and glutamine.^120^, ^138^	HIF1α represses the aspartate‐generating GOT1 and GOT2 enzymes reducing intracellular aspartate levels.^120^	Cancer cell proliferation can be driven by cellular aspartate, and HIF1α is a direct inhibitor of aspartate biosynthesis.^120^	Assess the specific changes of aspartate biosynthesis via stable isotope‐resolved metabolomics^120^
PDK	Glucose metabolism: PDK inhibition drives the shift from aerobic glycolysis to OXPHOS.^139^, ^140^, ^141^	Inhibition of PDKs increases OXPHOS by activating PDC.^139^, ^140^, ^141^	The application of PDK inhibitors, as a potential therapeutic, has seen widespread success in models of multiple cancers.	Metabolomic analysis via CE‐TOFMS to assess the level of intracellular metabolites playing a role in central carbon metabolism^142^
ACC	Fatty acid synthesis: Elevated de novo lipogenesis rates in the MASLD, leading to accumulation of triglycerides in the liver.^143^	Inhibition of ACC can reduce the synthesis of fatty acids and accelerate the reaction of cytoplasmic acetyl‐CoA carboxylation into malonyl‐CoA.^143^, ^144^, ^145^	ACC inhibitor has anticancer and antidiabetic effects, including Soraphen A, TOFA, and CP‐640186.^146^, ^147^, ^148^	Targeted metabolomics and stable isotope‐resolved metabolomics were combined to reveal the changes in metabolism^143^, ^144^, ^145^
IDH1	Energy metabolism: Produces the cancer metabolite 2‐HG.^149^	IDH1‐mut consumed NADPH and reduced α‐ketoglutarate to a novel oncometabolite 2‐HG.^149^, ^150^, ^151^	IDH1 mutations are especially prevalent in malignant gliomas.	Metabolomics analysis via CE‐TOFMS and LC‐TOFMS^149^, ^150^, ^151^, ^152^ and the lipidomics analysis via LC–MS^153^
LDH	Energy metabolism: Elevated levels of lactate and reduced levels of TCA cycle intermediates such as succinate and citrate in disease models (e.g., diabetes).^154^	LDH inhibition reduces the growth rate of glycolytic tumors and redirects pyruvate to support oxidative phosphorylation.^154^, ^155^, ^156^	Dependence of many cancers on aerobic glycolysis makes LDH a viable therapeutic target. Current LDH inhibitors include galflavin, oxalat,^157^ and FX11.^158^	^1^H NMR spectrometry; Metabolic effects due to regulation and inhibition of LDH revealed through metabolic networks^154^, ^155^, ^156^
GLS	Glutamine metabolism: Glutamine catabolism is an important marker of cancer metabolism,^159^ producing ATP, NADPH, and supporting the synthesis of nucleotides and lipids.^160^	Targeting GLS can disrupt glutamine metabolism, which is essential for the rapid proliferation of cancer cells.^161^	Gls‐dependent cancer cells demonstrate the potential of GLS as a therapeutic target. GLS inhibitor includes CB‐839^162^ and BPTES.^163^	Reveal the molecular mechanism of GLS activity and the targeted MoAs^159^, ^164^, ^165^ Metabolomics confirmed the biological efficacy of different inhibitors and revealed differences in target specificity.^161^

Abbreviations: 2‐HG, 2‐hydroxyglutaric acid; ACC, acetyl‐CoA carboxylase; FAAH, fatty acid amide hydrolase; GLS, glutaminase; HIF1α, hypoxia‐inducible factor‐1α; IDH1, isocitrate dehydrogenase 1; LDH, lactate dehydrogenase; NATs, N‐acyl taurines; OXPHOS, oxidative phosphorylation; PDC, pyruvate dehydrogenase complex; PDK, pyruvate dehydrogenase kinase.

### 4.2 Metabolomics facilitates to identify targets of small molecules

Small‐molecule drugs, while having broad applicability and therapeutic potential, present challenges due to their complex MoAs involving multiple targets. The binding of these small molecules to proteins can alter enzyme activity, resulting in abnormal metabolic product accumulation or depletion. To address these complexities, integrating metabolomics with bioinformatics allows for the monitoring of metabolic trends and the identification of key enzyme targets, supported by various metabolomic‐based approaches such as high‐throughput metabolomics, dose–response metabolomics, and stable isotope‐resolved metabolomics.

#### 4.2.1 Metabolomics contribute to target identification via bottom‐up approach

The introduction of exogenous compounds into cells can lead to competitive binding with specific protein receptors, some of which can catalyze the metabolite transformations. As a result, the downstream changes in the metabolome provide insights into the enzyme targets that interacted with these compounds. This concept underpins the metabolomics‐driven “bottom‐up” approach for identifying drug targets, enabling the discovery of enzyme targets and clarifying the MoAs of drugs through the analysis of metabolic pathway alterations.^166^ For example, Zhao et al.^167^ analyzed the *db/db* mice samples (e.g., urine, serum, and kidney) following acteoside administration via UHPLC–LTQ–orbitrap MS‐based nontargeted metabolomics. They found that the dysregulation of amino acid metabolism due to diabetes can be reversed by drug (ACT), such as restoring phenylalanine levels. Combined with network pharmacology, drug targets such as AKT2 and AKT1 were discovered.^167^ Zhou et al.^168^ employed targeted metabolomics to analyze the metabolic profile of elemene treatment on PC3 prostate cancer cells. Changes were observed in circulating metabolic intermediates within glycolysis and the TCA cycle, which further validated that 6‐phosphofructo‐2‐kinase and fructose‐2,6‐biphosphatase 3 are critical targets in mediating the elemene‐induced inhibition of glycolysis in prostate cancer cells.^168^ Similarly, Liu et al.^169^ conducted metabolomic analysis on Tao‐Hong‐Si‐Wu decoction extract and treated mouse tissue samples, identifying 11 key metabolites. Integrated with network pharmacology analysis followed by relevant experimental validation, the study predicted Vegfa, IL‐6, and TNF genes as the primary targets of Tao‐Hong‐Si‐Wu decoction in treating sepsis.^169^

**FIGURE 3:**
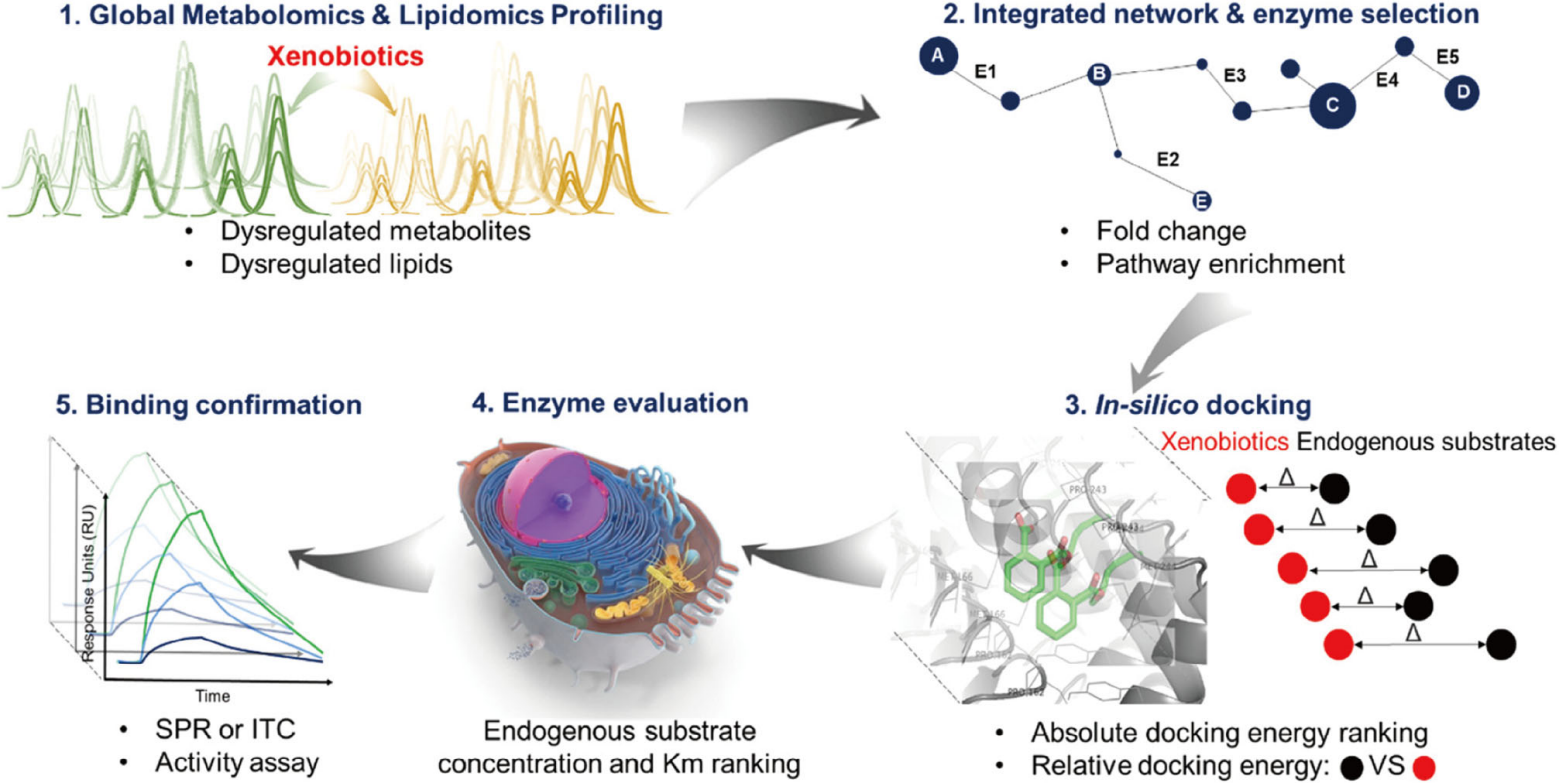
Schematic diagram of the bottom‐up strategy for the discovery of novel molecular targets. Interaction networks were established using comprehensive metabolomics analyses to identify key pathways and metabolic enzymes, then validating the accuracy of enzyme targets. Reprinted with permission from Ref. 166 Copyright 2024 American Chemical Society.

In our group, we used a bottom‐up approach to study triphenyl phosphate (TPhP), employing metabolomics and lipidomics to screen dysregulated metabolites and lipids (Figure 3). Using UHPLC–Q‐TOF–MS, we identified 72 dysregulated metabolites, highlighting key metabolic pathways through the construction of an interaction network by Metscape.^166^ Subsequently, molecular docking and surface plasma resonance analysis validated TPhP's interaction with enzymes in the β‐fatty acid oxidation pathway. A series of validation experiments confirmed the interaction of TPhP with hydroxyl‐coA dehydrogenase and hydroxysteroid 17‐beta dehydrogenase 10 (HSD17B10).^166^ This approach demonstrates the efficiency and cost effectiveness of metabolomics in large‐scale target discovery.

#### 4.2.2 Dose–response metabolomics contribute to target identification

The therapeutic efficacy and safety of a drug are inherently dose dependent, as both beneficial and adverse responses vary with drug concentrations.^170^, ^171^ Relying on a single‐dose metabolomics study may overlook critical metabolic responses that occur at other dose levels. For instance, while acetaminophen is effective at therapeutic doses, higher exposures can deplete GSH, induce oxidative stress and mitochondrial dysfunction, and lead to hepatotoxicity.^172^ Multidose metabolomics addresses these limitations by providing a comprehensive assessment of metabolic changes across various drug doses, allowing for a deeper understanding of MoAs and potential off‐target effects. Conventional metabolomics may be insufficient for the evaluation of chemicals that could potentially affect multiple proteins with varying potencies.^173^, ^174^ Therefore, dose–response metabolomics has emerged as a powerful method to quantify drug concentration effects, elucidate metabolic pathways, and identify therapeutic targets.

Dose–response metabolomics quantifies the relationship between drug concentration and its effects on metabolites within a sample,^174^ allowing researchers to construct dose–response curves that clarify potential dose‐dependent metabolic responses and provide vital insights into MoAs of drugs and possible off‐target effects. For example, Patti et al.^174^ conducted dose‐effect curve modeling to find 908 features exhibiting statistically significant monotonic rises or declines in the medication etomoxir. The conclusion that at least two separate targets for etomoxir was inferred by analysis of these dose‐dependent metabolite alterations using three‐dimensional (3D) PCA, which offers potential explanations for its hepatotoxicity.^174^ Similarly, Pink et al.^175^ characterized the dose–response relationship of 3‐nitrobenzanthrone (3‐NBA) in a human urothelial carcinoma cell line. At low doses, the cells activated adaptive mechanisms and elevated antioxidant levels in response to the toxic 3‐aminobenzanthrone metabolite derived from 3‐NBA. At higher doses, cellular adaptation decreased, leading to a greater reliance on metabolic reprogramming to sustain homeostasis, which resulted in increased oxidative stress, reduced proliferation, and mitochondrial hyperpolarization, with significant involvement from the pentose phosphate pathway and folate metabolism.^175^ In summary, dose–response metabolomics effectively elucidates the intricate connections between drug exposure, metabolic alterations, and potential therapeutic or off‐target effects, thereby offering critical insights for drug development and optimization.

**FIGURE 4:**
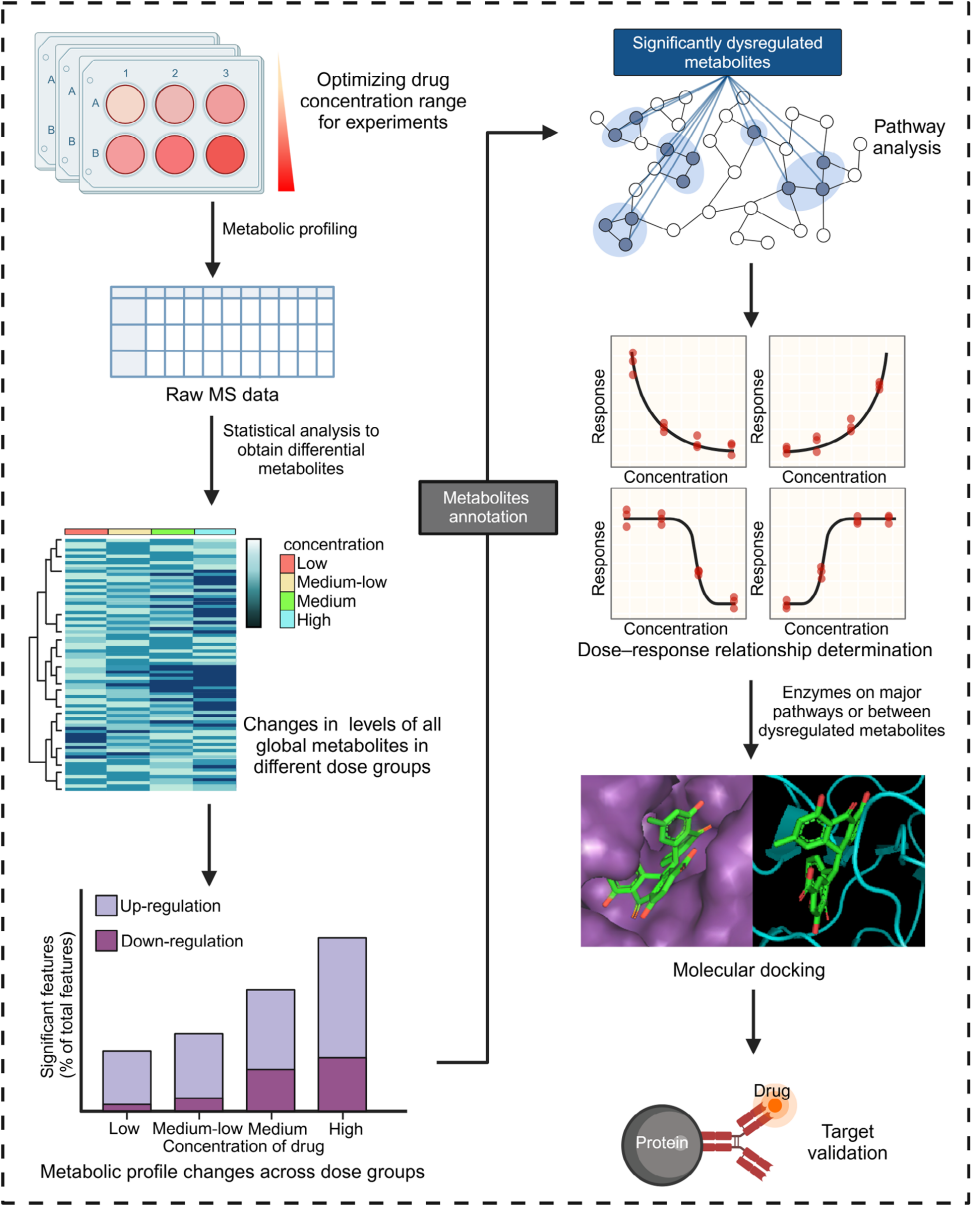
Dose–response metabolomics target identification flowchart. Dose–response metabolomics facilitates the identification of key metabolic targets by systematically analyzing metabolite changes across varying doses. This approach underscores the dose‐dependent effects on metabolic responses, offering valuable insights into regulatory mechanisms within biological systems (created by Biorender; https://biorender.com).

The identification of drug targets through dose–response metabolomics involves a systematic four‐stage process (Figure 4): (1) *Metabolomic analysis at various doses*. The initial step encompasses the determination of an appropriate dose gradient for drug administration through pre‐experimentation. This step established a foundation for the subsequent construction of dose–response curves. Metabolomic analyses of the distinct dose groups are typically conducted using LC–MS techniques. (2) *Construction of dose–response curves*. The large nontargeted metabolomics datasets pose computational challenges. To extract and categorize the dose–response curves for each measured metabolic feature, specialized software tools, such as TOXcms were used.^176^ The dose–response curves can be classified into four main response trends: “no difference,” “increase,” “decrease,” and “inflection” (further divided into V‐shaped and inverted V‐shaped).^174^ Based on TOXcms, Fang et al.^176^ identified and obtained 29 significantly dysregulated metabolites of bisphenol A (BPA) exposure to cancer cell lines and concluded that the purine metabolism is the most important target pathway of BPA. (3) *Identification of key target proteins*. These dysregulated metabolic characteristics exhibit a clear dose–response relationship. These features can be used to identify key metabolites and enzymes related to the mechanism of drug action. Then schematic indications of the potential number of targeted proteins can be provided based on the response trends and effective dose (ED_50_) values.^177^ (4) *Experimental validation*. The correctness of the identified targets is subsequently verified through a combination of molecular docking studies, gene knockout experiments, and animal models to confirm the predicted drug–target interactions and their functional consequences. This systematic, multistep approach enables researchers to utilize dose–response metabolomics to uncover the molecular targets underlying the observed phenotypic changes induced by drug treatment.

The application of dose–response metabolomics extends beyond the investigation of individual compounds, demonstrating its versatility in the identification of targets within complex mixtures, such as TCMs and environmental exposures.^178^ Our group conducted a dose–response metabolomics of the TCM *Venenum Bufonis* (VBF), known for its significant anticancer properties, demonstrated a dose‐dependent decrease in DNA synthesis‐related metabolites such as UTP, ATP, GTP, CTP, and UDP, suggesting that VBF primarily targets the mTOR pathway to inhibit both salvage and de novo pyrimidine and purine synthesis, ultimately leading to cell cycle arrest.^179^ Colas et al.^180^ investigated the alterations in river biofilms following cobalt (Co) exposure, identifying two primary concentration ranges of metabolic responses. The CRIDeR stage exhibits a biphasic trend indicative of a defensive response, while the CRIDaR stage exhibits a monotonic trend associated with cellular damage and metabolic dysregulation. The authors further elucidated the dose‐dependent changes in the activities of metabolic enzymes, such as catalase and superoxide dismutase, during the CRIDeR stage, revealing the underlying defense mechanisms against the elevation in intracellular Co content.^180^ Similarly, Lu et al.^181^ investigated the microstructural and physiological characteristics of biofilms exposed to different concentrations of Mg^2+^. The research shows that Mg^2+^ significantly affects biofilm formation in a concentration‐dependent manner by regulating the microstructure of *Escherichia coli* and inducing metabolic modifications in nucleotide and amino acid metabolism.^181^ These studies collectively illustrate the application potential of dose–response metabolomics in the multitarget screening of TCMs, environmental exposures, and other complex mixtures, providing valuable insights into their MoA and therapeutic potential.

#### 4.2.3 Stable isotope‐resolved metabolomics contribute to the target identification

The inherent complexity of metabolic networks can limit the accurate representation of metabolic activity, even though standard metabolomics provides valuable insights into static metabolite abundance.^182^ Metabolite accumulation may result from increased production or decreased consumption. The actual metabolic fluxes and the contribution of substrates may differ under the intertwining of multiple pathways, despite the same level of intermediate metabolites.^26^ In contrast, metabolic flux analysis (MFA), enabled by stable isotope‐resolved metabolomics (SIRM), offers a more comprehensive understanding of metabolic regulation by directly measuring metabolic reaction rates (fluxes).^183^ Pertinent biomarkers and therapeutic targets can be determined by accurately identifying and comprehending the primary metabolic pathways that are involved in drug treatment. This will provide a scientific basis for clinical diagnosis and drug–target therapy. MFA enables the observation of metabolic activity through dynamic means, thus offering a more comprehensive and in‐depth understanding of the mechanisms and roles of metabolic regulation.^184^

The utilization of isotope tracing technology in metabolic studies has evolved over the decades.^185^ With researchers transitioning from the use of radioactive tracers to the safer application of stable isotopes, such as ^13^C, ^2^H, and ^18^O,^187^, ^188^, ^189^ these isotopic labels are used to track metabolic pathways at varying labeling ratios. The quantification of isotope enrichment in cellular metabolites (M+0/M+1/M+2/…) to assess metabolic activity.^190^, ^191^, ^192^ By integrating experimental data from isotope labeling^193^ with metabolic network models,^194^ researchers can mathematically determine metabolic fluxes and analyze the changes as well as distribution of compounds in metabolic pathways. Thereby, the understanding of metabolic reprogramming can be advanced. Metabolic network models are typically associated with searchable and queryable databases, such as KEGG, Virtual Metabolic Human, and MetaCyc.^183^ Two common experimental designs, stationary and nonstationary MFA (i.e., isotopically nonstationary MFA), are utilized in MFA.^183^, ^195^, ^196^, ^197^ Among these techniques, ^13^C‐MFA is considered the primary technique for determining intracellular fluxes. It offers a significant advantage in enhancing flux accuracy due to the substantial redundancy constraints provided for flux estimation.^194^, ^198^ Additionally, various software platforms, including eiFlux, INCA, OpenMebius, NSMFRA, ScalaFlux, and KFP, are available for flux analysis.^199^, ^200^, ^201^, ^202^ For instance, Zou et al.^203^ utilized ^13^C‐labeled Gln for metabolic tracking, analyzed metabolic fluxes in vitro and in vivo, and combined with transcriptomics to uncover key reprogrammed metabolic pathways in the fibrotic microenvironment. McConville et al.^204^ utilized multiple ^13^C‐labeling to detect atypical metabolic pathways in *Plasmodium falciparum*, highlighting the activity of its metabolite repair pathway and providing new research directions for drug discovery.^204^ The continued advancement and application of isotope tracing technology have enabled researchers to unravel the complex mechanisms underlying metabolic reprogramming, paving the way for more comprehensive target identification in biological systems.

**FIGURE 5:**
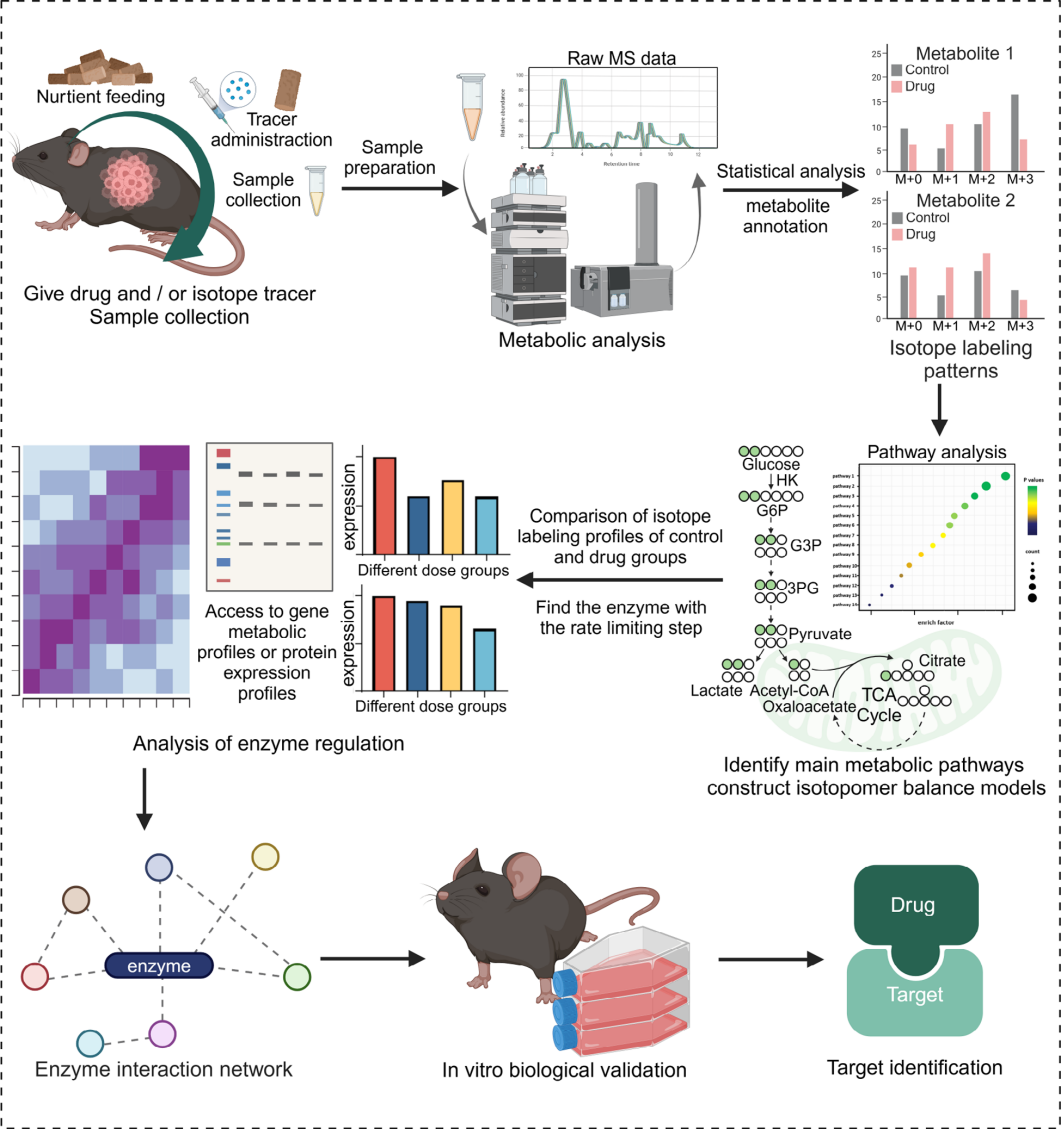
Metabolic flux analysis (MFA) provides highly informative information in the identification of drug targets. MFA allows for real‐time tracking of metabolite flow and transformation by incorporating labeled isotopes. This technique uncovers the dynamic changes in metabolic pathways, offering a unique perspective on the intracellular metabolic network and biological response mechanisms (created by Biorender; https://biorender.com).

MFA provides a more precise assessment of metabolic pathway activity, making it an essential tool for identifying key molecules as potential drug targets for further validation (Figure 5). Locasale et al.^205^ elucidated the variable mitochondrial metabolism subsequent to drug administration by scrutinizing the metabolic profiles of the drug treatment group. Through the collective use of isotopically labeled substrates, including U‐^13^C glucose, U‐^13^C palmitate, and U‐^13^C Gln, the authors traced the alterations in mitochondrial substrate utilization patterns upon metformin treatment. Their results demonstrated that metformin induced changes in substrate utilization within mitochondria and anabolic processes. These alternation impact heightened levels of tricarboxylic acid (TCA) cycle intermediates, NADH accumulation, and the generation of ROS.^205^ This MFA‐based approach suggested the mitochondrial metabolic targets underlying the anticancer activity of metformin and the factors influencing metformin resistance. Similarly, Tian et al.^206^ used SIRM to reveal multiple targets of Xiaoyaosan for improving TCA cycle disorders. The decrease in isotopic abundance of key metabolites, such as citric acid and glutamate, at M+0 indicated the role of XYS in repairing the TCA cycle. Furthermore, the isotopic abundance of metabolites in the TCA cycle at M+2 and M+3 pointed to the critical involvement of enzymes like pyruvate carboxylase and pyruvate dehydrogenase in regulating the entry of pyruvate into the TCA cycle.^205^, ^206^, ^207^ In another example, Lyssiotis et al.^207^ found that glucose and uridine promote metabolism to a similar extent, confirming the catabolism of pancreatic ductal adenocarcinoma to uridine in vivo due to the high uridine, UMP, and UTP labeling (M+5) by SIRM analyses. This suggested the potential of uridine phosphorylase 1 (UPP1) as a new target for cancer therapy. Additionally, DeBerardinis et al.^208^ employed ^15^NH_4_^+^ labeling to demonstrate that the knockout of carbamoyl phosphate synthetase‐1 (CPS1) reduced the isotope abundance of thymidine at M+1, revealing the critical involvement of CPS1 in thymine synthesis and the dependence of non‐small‐cell lung cancer genotypes on pyrimidine metabolism. These examples illustrate the power of MFA and SIRM in elucidating the dynamic alterations in metabolic pathways upon various treatments. So that they could identify key metabolic targets for drug development and therapeutic interventions.

#### 4.2.4 High‐throughput metabolomics contributes to the target identification

Metabolomics presents a cost effective and easily implemented approach to detect thousands of metabolite components in samples, offering significant advantages in the analysis of large numbers of samples.^209^ Metabolomics allows for the direct detection of drug‐induced metabolic changes associated with phenotypic effects,^210^ driving the demand for high‐throughput methods in multidose studies, active molecule screening, and the exploration of individual differences in drug metabolism.^209^ Traditional MS‐based metabolomics face limitations due to chromatographic separation speed, which determines the time required for sample detection. However, the development of higher‐throughput LC–MS and CE–MS technologies has significantly enhanced analysis efficiency. Advancements in LC–MS, such as ultra‐performance LC (UHPLC), optimized chromatography columns, have significantly improved analysis efficiency by reducing solvent consumption^211^ and analytical cycle times, enabling large‐scale metabolic phenotyping.^212^ For example, Rainville et al.^213^ achieved human urine analysis in just 3 min using UHPLC–MS, detecting 10,400 features. While Link's team quantified primary metabolites in under 2 min with a short column and a sensitive QqQ instrument.^214^ Higher‐throughput LC–MS methods may sacrifice peak capacity and total feature detection, but incorporating ion mobility (IM) can mitigate these issues.^212^ Alternative strategies like DI and flow injection analysis (FIA) further enhance throughput, which abandon chromatographic separation.^210^, ^215^ DIMS analysis can be up to 20 times faster than LC–MS, though it may require additional cleaning steps to address system contamination and sample carryover.^216^, ^217^, ^218^ FIA allows for the injection of 1000–2000 samples per day, with small injection volumes and short reaction times, which also elevate the analysis range and sensitivity, enabling high‐throughput detection.^210^, ^219^, ^220^ Innovations like flow injection time‐of‐flight MS (FIA TOF MS) have further reduced analysis time to under 1 min, enabling the analysis of over 1400 samples per day.^221^ Additionally, high‐throughput NMR‐based metabolomics offers simple sample preparation and broad metabolite detection capabilities.^222^ The introduction of higher‐density microplates and droplet microarrays,^223^, ^224^, ^225^ along with advances in miniaturization and automation,^226^, ^227^ has solidified high‐throughput metabolomics as a crucial tool in drug discovery and development.

High‐throughput metabolomics provides an efficient method for rapid identification of metabolic targets and reactions. It enables us to move beyond the traditional bottom‐up approach that focus on complex metabolite changes, driving researchers to develop innovative experimental designs like high‐throughput screening (HTS) for discovering new drugs and uncovering unknown enzyme functions, including applications in in vitro toxicology. Clare et al.^228^ conducted HTS using a 384‐well format to evaluate five types of snake venoms, identifying four snake venom metalloproteinase inhibitors and two lead compounds. Sauer et al.^229^ characterized the functions of 1725 overexpressed or purified *E. coli* proteins by monitoring changes in metabolite abundance in supplemented metabolome extracts, which revealed potential enzyme catalysis; they established evaluation criteria based on empirical *Z*‐score thresholds derived from prior analyses of 189 known enzymes, ultimately identifying 241 potential new enzymes.^229^ Subsequent experiments successfully confirmed 12 of these newly identified enzymes,^229^ suggesting this approach could aid in discovering drug target information by identifying affected metabolic enzymes.

**FIGURE 6:**
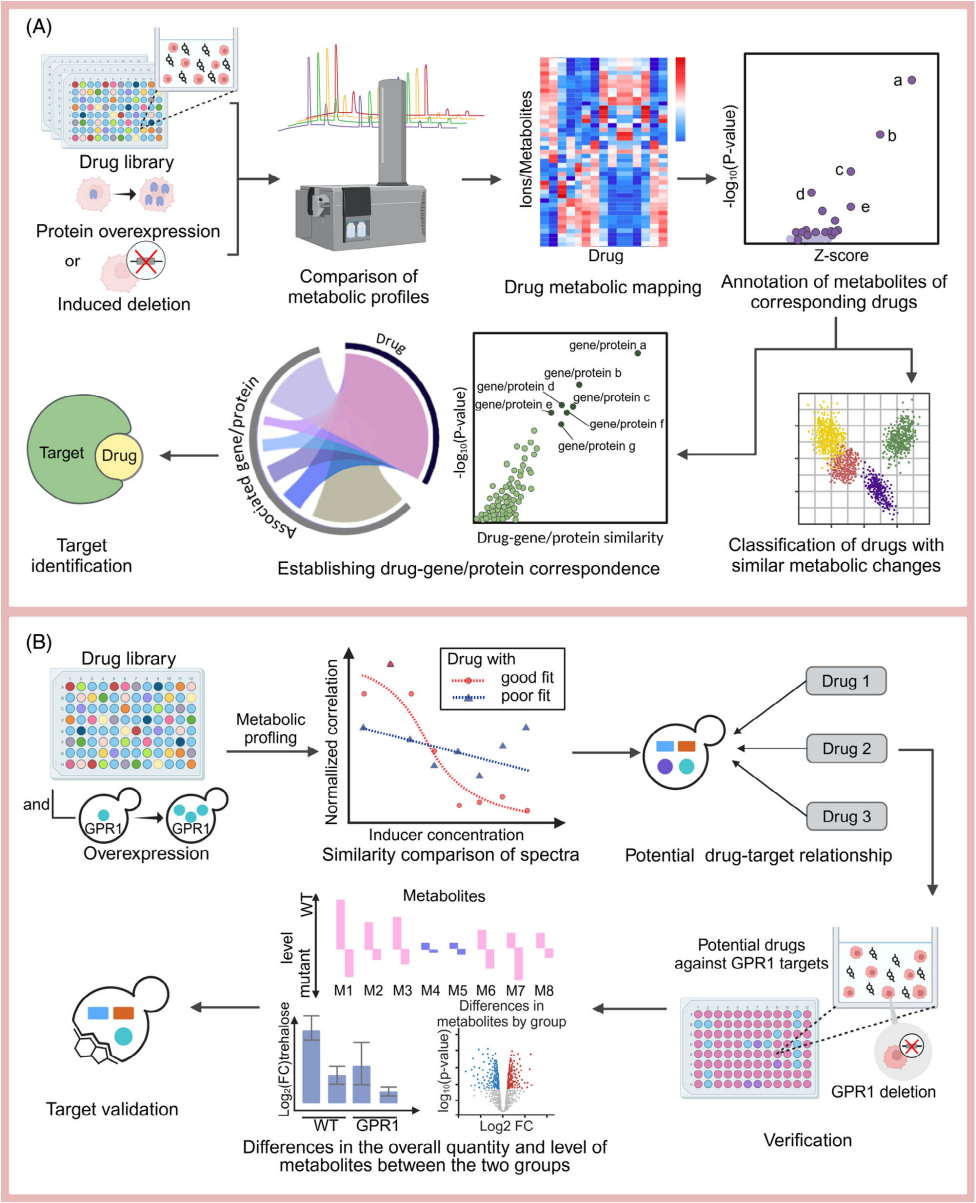
High‐throughput metabolomics facilitate target identification, which is particularly suitable for large‐scale sample screening. (A) General process of target identification by high‐throughput metabolomics. (B) Specific example (GPR1) of target identification by high‐throughput metabolomics (created by Biorender; https://biorender.com).

A key application of high‐throughput metabolomics is HTS utilizing drug libraries (Figure 6A), which distinguishes itself from conventional methods by using the metabolome as a direct indicator of cellular biochemistry, thereby minimizing metabolism‐related issues for screened drugs.^230^ By comparing spectral similarities, high‐throughput metabolomics can predict drug–target relationships across large batches. For instance, Zampieri et al.^231^ developed a method that integrates experimental calculations with metabolic analyses of drug responses and the metabolic and chemical genome profiles of single‐gene deletion strains. This approach allows for the classification of drugs according to their metabolomes and MoAs based on the screening results. Additionally, a weighted scoring function is employed to correlate enzymes with metabolic changes, subsequently linking genes to construct drug–target relationships. Similarly, Sauer et al.^232^ utilized high‐throughput metabolomics to compare the metabolomic profiles of compound treatments with those from induced membrane protein overexpression in yeast cells, identifying several new targets, including ibuprofen's effect on GPR1 signaling (Figure 6B).

High‐throughput metabolomics enhances sample analysis efficiency by optimizing instrumentation and reducing sample volume requirements, allowing researchers to analyze thousands of samples, a growing standard in the pharmaceutical industry that necessitates more efficient throughput methods. Zamboni et al.^210^ propose that efficiency can be significantly enhanced without compromising sensitivity by optimizing the dead time between injections. Advances in injection techniques, such as droplet‐based microfluidics and surface acoustic sampling, have made it possible to analyze more than 10,000 samples per day.^210^ For example, Kim et al.^233^ describe an innovative analytical method that integrates time‐of‐flight secondary ion MS (ToF‐SIMS) with a microfluidic cell system, referred to as the system for analysis at the liquid vacuum interface). They introduce a rapid, high‐throughput platform for the quantitative analysis of amino acids in matrix deposits, which are formed from freeze‐dried solution drops through ice sublimation on a parylene film microarray substrate.^234^ However, the drive for higher throughput necessitates the sacrifice of the separation of compounds with mass‐to‐charge ratios (e.g., isomers).^234^ This challenge demands not only higher resolution but also more advanced analytical methods. It is crucial to exclude the influence of the matrix effect and accurately identify key metabolite characteristics from complex signal peaks. According to the study by Zamboni et al.,^210^ the former issue can be addressed through effective metabolite extraction protocols that ensure a constant concentration of analytes and sample clean‐up. While the latter urgently needs the development of a new computing method for data integration.

#### 4.2.5 Multiomics contributes to targets identification

Metabolomics currently faces several limitations, including limited coverage of metabolites, lack of annotations for specific compounds, and potential degradation of active metabolites, which can obscure critical metabolites and pathways, hindering a comprehensive understanding of biological mechanisms. Single‐omics approaches also typically capture only a small subset of biological changes,^235^ making the integration of multiomics data a powerful strategy to address these challenges.^236^ By elucidating the origin and changes of metabolites, transcriptomics and genomics enhance the understanding of metabolic regulation at the genetic level to complement metabolomics. While proteomics reveals the relationships between metabolites and proteins through the analysis of protein expression and activity. Additionally, epigenomics helps identify epigenetic factors that influence metabolic levels.

Integrating metabolomics with other omics provides researchers with a comprehensive and systematic understanding of disease mechanisms and MoAs of drugs, thereby improving the predictive accuracy of therapeutic targets' roles and clinical effects.^237^ For example, Jiang et al. categorized triple‐negative breast cancer (TNBC) into three distinct subtypes based on metabolic characteristics identified in the polar metabolome and lipid group. This metabolomics‐driven classification offered more insights compared with transcriptome‐based subtype categorization. Concurrently, genomics was employed to explore potential genomic drivers underlying metabolomic characteristics. By associating the metabolome with the genome, key metabolic genes were identified, and the accuracy of classification was validated. This multiomics approach not only revealed metabolic heterogeneity within TNBC but also enabled more precise target identification and understanding of the underlying causes of phenotypic variability in complex diseases such as chronic kidney disease and idiopathic pulmonary fibrosis.^238^ In another example, Cai et al.^239^ combined metabolomics with chemical proteomics and revealed that acetyl‐CoA carboxylase 1 and acetyl‐CoA carboxylase 2 are the direct molecular targets for perfluorooctanoate. While Jiang et al.^240^ demonstrated the neuroprotective effects of scutellarin via targeted metabolomics and 13C‐MFA, highlighting the role of pyruvate dehydrogenase kinase 2 (PDK2) in mitochondrial glucose oxidation. Additionally, our group's research identified SOD3 and GPX4 as novel targets for hepatotoxicity induced by Benzo[α]pyrene through a combination of metabolomics and chemical proteomics.^241^

Integrating metabolomics with other omics strategies has significantly improved the understanding of drug repositioning opportunities through new target identification. For example, Zou et al.^242^ conducted a metabolomics analysis of metformin, which has shown remarkable efficacy in inhibiting tumor growth, revealing significant changes in metabolic pathways such as glutathione metabolism and polyamine synthesis in thyroid cancer. By integrating transcriptomics data, the researchers confirmed that metformin inhibited glycolysis by downregulating key glycolytic enzymes while upregulating the expression of isocitrate dehydrogenase, thereby exerting anticancer effects. Additionally, metabolomics revealed reduced levels of short‐chain acylcarnitine in tumor tissues of metformin‐treated patients, indicating that metformin inhibits fatty acid oxidation, likely through direct effect on mitochondrial complex I.^243^ Multiomics strategies also pave the way for combination therapy. For instance, Rosner et al.^244^ found that BACH1 deficiency in TNBC cells promotes mitochondrial respiration, and subsequent analyses suggested that combining metformin, a mitochondrial respiratory inhibitor, could enhance this effect. Overall, compared with single metabolomics, multiomics analysis construct a dynamic multidimensional networks of biochemical interactions rather than static maps.^245^ Overall, multiomics analysis creates dynamic, multidimensional networks of biochemical interactions rather than static maps, allowing researchers to gain a comprehensive understanding of drug effects, identify novel therapeutic targets, and explore rational combination therapies, thus advancing drug repositioning and development efforts.

Multiomics strategies indeed present unique challenges, particularly in sample collection and processing, as the response times and lifetimes of biomolecules can vary greatly within and across different omics layers. Determining the optimal sampling time to account for these variations is critical.^235^ Additionally, advancements in MS imaging and single‐cell technologies highlight the impact of tissue and cellular heterogeneity on experimental outcomes. Developing a well‐considered sample extraction and distribution scheme is essential for ensuring even representation and accurate analysis across multiple omics layers. For example, tissues may be homogenized or sectioned to provide samples for various analyses. When working with limited samples, separate extractions may yield inadequate sample sizes.

Integrate multiple omics data can enhance comprehensive analysis through two main methods: conceptual and statistical integration. Conceptual integration analyzes each omics dataset separately and synthesizes the results, similar to a 1+1 approach.^246^ Statistical integration (synchronization strategy), however, involves simultaneous analysis of data from multiple omics layers, revealing associations between features and providing a more comprehensive view.^247^ This method amplifies relationships and changes that may be less evident in single‐omics analyses. The *synchronization strategy* for multiomics integration faces three major computational challenges: (1) correlating and tracking molecular components across different layers and databases; (2) ensuring reliable data normalization and conducting multilevel data analysis; and (3) effectively visualizing the results.^248^ In multiomics strategies centered on metabolomics, the goal is to integrate data from other omics layers into a metabolic network model, allowing exploration of related genes, proteins, and differential metabolites. Achieving this necessitates ongoing advancements in fields like computer vision and natural language processing to support unbiased integration of high‐dimensional data.^249^ Relevant data integration methods and application tools have been detailed in previous reviews (e.g., Refs. ^235^, ^248^, ^249^, ^250^) and will not be further discussed here. However, there remains significant potential for improvement, particularly in addressing objective differences caused by external factors (e.g., batch effects).^250^

### 4.3 New developments of metabolomics to identify small molecule targets

#### 4.3.1 MSI contributes to small molecule target identification

MSI is a crucial technique in spatial omics, providing a detailed view of metabolite distribution and helping elucidate their regulatory mechanisms. As a powerful analytical tool, MSI ionizes individual pixels from biological samples, generating mass spectral maps that are reconstructed into high‐resolution images.^251^ This method directly captures the spatial distribution of molecules in thin tissue sections, with spatial resolutions ranging from 10 to 200 µm.^252^ MSI effectively reveals molecular heterogeneity across biomolecules, including proteins, peptides, lipids, polysaccharides, amino acids, and oligonucleotides.^253^, ^254^

Matrix‐assisted laser desorption/ionization (MALDI) has been the predominant MSI method since its introduction in 1994. MALDI uses a laser‐absorbing matrix to interact with analytes, forming cocrystals that enhance imaging and detection.^255^, ^256^ The ionization process, triggered by laser irradiation of tissue sections, ejects compounds for subsequent mass spectrometric analysis.^257^ More recently, desorption electrospray ionization (DESI), introduced in 2007, has enabled direct analysis of living surfaces or tissue sections without prior sample preparation.^251^, ^252^, ^258^ Advances in spatial resolution and instrument sensitivity have focused on improving ionization, transmission, and detection efficiencies.^259^ MALDI–MSI typically achieves moderate spatial resolution (5–50 µm) in a nontargeted manner.^260^ While the SpaceM approach, integrating MALDI–MSI with optical microscopy and digital image processing, facilitates the correlation of metabolite data with the cells of origin.^261^ The development of MALDI‐2 has improved resolution to 600 nm by enhancing the ionization efficiency,^262^, ^263^ allowing for single‐cell analysis.^258^, ^259^ Other techniques like air‐flow assisted desorption electrospray ionization (AFADESI–MSI)^264^, ^265^ and surface‐assisted laser desorption/ionization (SALDI–MSI)^266^ have further expanded MSI applications, enhancing ionization efficiency and reducing background noise. Recent advancements, such as 3D MSI, have extended spatial resolution to include both lateral and depth dimensions.^259^ Several comprehensive reviews detail these technological advances, applications, and operational principles.^252^, ^256^, ^258^, ^267^, ^268^, ^269^, ^270^

MSI is especially valuable in revealing metabolite discrepancies across various spatial contexts, making it a promising tool for target discovery in clinical metabolomics. It provides high coverage, sensitivity, throughput, reproducibility, and nonspecificity, allowing spatial analysis of highly complex biological samples.^266^, ^267^ In contrast, conventional metabolomics often overlooks spatial distribution within tissues, limiting insights into metabolic heterogeneity, such as in tumor environments.^253^ MSI's spatial metabolomics capabilities allow for the investigation of chemical distribution across different scales, illuminating complex biological systems and aiding in the understanding of drug mechanisms and therapeutic predictions. For instance, the different metabolic activity between the external regions and the center in esophageal cancer multicellular tumor spheroids^271^ and the heterogeneous distribution of active metabolites of gemcitabine in pancreatic cancer tumor tissues^272^ illustrates the value of spatial metabolomics in providing insights into complex biological systems.

**FIGURE 7:**
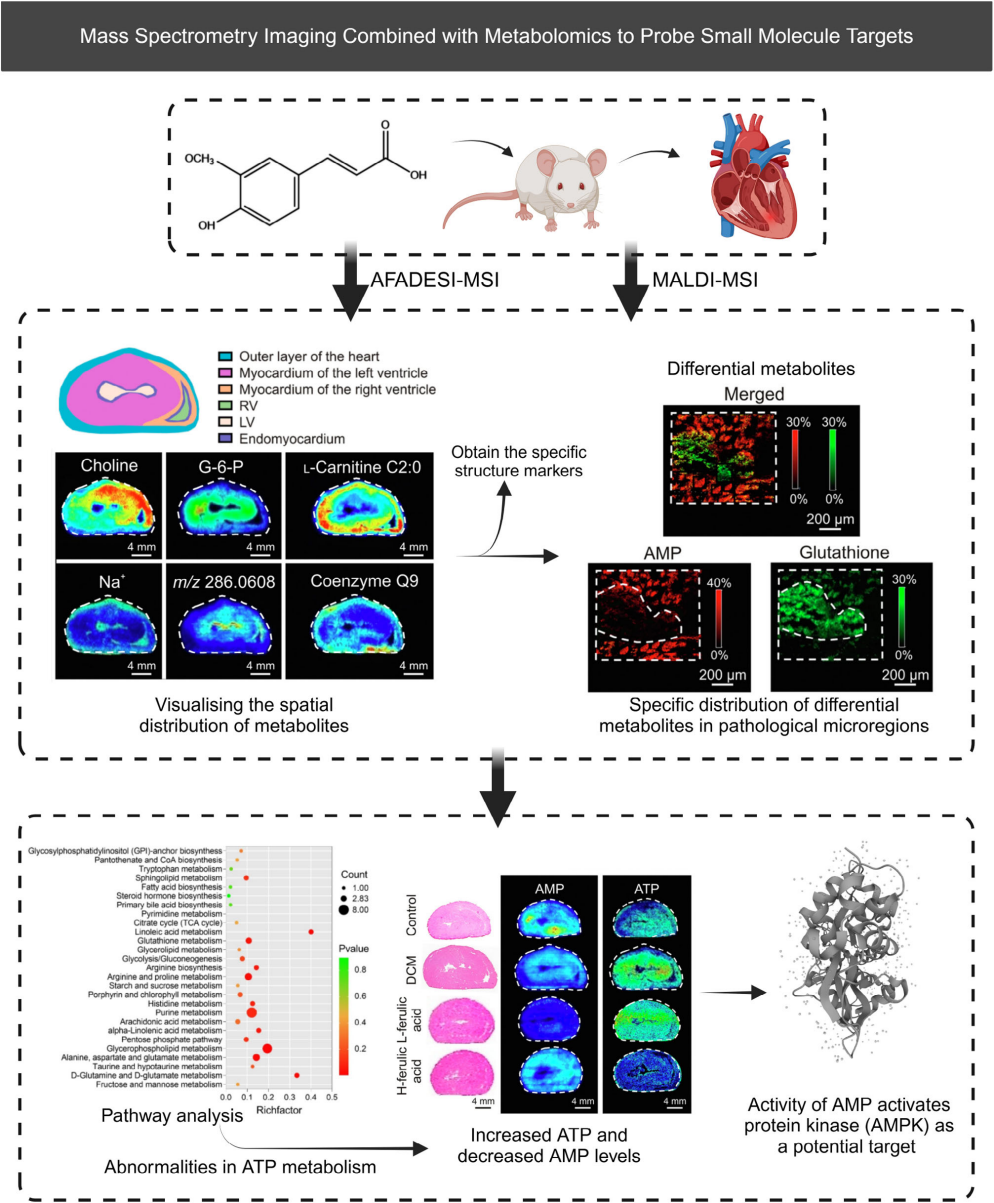
Mass spectrometry imaging (MSI) combined with metabolomics to probe small molecule targets. It allows visualization of the spatial distribution of metabolites and further screening for differential metabolites associated with pathological regions (reprinted with permission,^273^ and then create overall flow chart by Biorender; https://biorender.com).

MSI has proven useful in studying metabolic reprogramming linked to specific structures, offering pathways for target identification.^102^, ^268^ For instance, Abliz et al.^273^ used MSI to uncover metabolic heterogeneity in a diabetic cardiomyopathy (DCM) model, identifying hundreds of metabolites distributed within the heart via AFADESI–MSI and MALDI–MSI analyses (Figure 7). They focused on fatty acid metabolism, oxidative stress pathways, and ATP metabolism due to the significant accumulation of differential metabolites in the lesion area.^273^ These heterogeneous distributions of metabolites suggest their role in maintaining the unique structural and functional characteristics of the cardiac region and may also serve as specific structural markers to guide investigations into localized metabolic activity. The findings suggested metabolic markers linked to lesion areas and potential therapeutic interventions, such as the reversal of DCM symptoms with ferulic acid through inhibiting AMPK activity. Supporting literature indicates that ferulic acid‐mediated AMPK activation improves cardiac function, reduces oxidative stress, and prevents myocardial damage in diabetic hearts through its anti‐inflammatory and antioxidant activities.

Similarly, AFADESI–MSI has identified abnormally expressed enzymes in esophageal squamous cell carcinoma based on the spatial discrepancies in metabolites. Abliz group revealed metabolic vulnerabilities that could be targeted for therapy, such as targeting enzymes pyrrolidine‐5‐carboxylate reductase 2 and uridine phosphorylase 1.^274^ The advantage of this approach lies not only in its high‐throughput characterization but also in the fact that it does not require the definition of a specific target of interest beforehand. Abliz et al.^275^ also mapped carnitine metabolism reprogramming in breast cancer, uncovering novel findings regarding the aberrant expression of carnitine palmitoyltransferase 2 and carnitine/acylcarnitine translocase. They also employed MSI to map the microregional distribution patterns of the sedative‐hypnotic drug candidate YZG‐331 in brain tissue sections, revealing its prominent distribution in the pineal gland and suggesting glutamate decarboxylase as a potential drug target.^276^ Sun et al.^277^ employed MALDI–MSI combined with IM to establish a high dimensionality reduction and spatial clustering method, effectively identifying the pathological features of advanced pulmonary fibrosis, including the specific accumulation of glycogen in myofibroblasts. They ultimately revealed glycogen as a promising target for pulmonary fibrosis. Spengler et al. introduced a high‐resolution (HR2) MSI method, which demonstrated an enrichment of the anticancer drug imatinib and isocyclophosphamide in the kidney, hinting at the location of potential drug targets.^278^

A novel target discovery approach combines spatiotemporal metabolomics with isotope tracing, addressing the limitations of traditional metabolic flux technologies.^279^ This method identifies drug‐related metabolic changes and interference points in metabolic pathways. For instance, Davidson et al.^280^ employed spatially resolved isotope tracing to elucidate metabolic activities in different tissues, which revealed regional differences in nitrogen source utilization for brain synthesis of glutamate. Patti et al.^281^ employed this integrated approach to investigate metabolite abundance and nutrient contribution in the brains of GL261 glioma mice. They also developed a spatial isotopologue spectral analysis method to evaluate the spatial distribution of metabolic turnover fluxes.^281^ Furthermore, future research will likely focus on integrating spatial metabolomics with multiomics strategies and computational advancements.^282^ He et al.^283^ demonstrated the potential of this integration by visualizing cellular and metabolic features in gastric cancer, identifying metabolic remodeling and interactions that could serve as therapeutic targets, including arginine deiminase or arginase.

In conclusion, MSI's ability to elucidate metabolite discrepancies across spatial contexts offers significant potential for advancing target discovery and clinical applications in metabolomics.

#### 4.3.2 Single‐cell metabolomics in identifying small molecule targets

Cells serve as the fundamental units of life, performing diverse functions essential for maintaining vital processes and transmitting genetic information in living organisms. While seemingly identical, cells can exhibit significant heterogeneity in their gene expression patterns and phenotypes.^284^, ^285^ This cellular heterogeneity unveils intricate biological phenomena, such as tumor heterogeneity.^286^, ^287^ Cancer stem cells (CSCs) play a key role in tumor heterogeneity, driving tumor growth, proliferation, metastasis, and resistance to therapy,^288^, ^289^ such as the varied expression of the AC133 epitope in colonic CSCs.^290^ Similarly, metabolic differences have been observed between prostate cancer cell lines, PC3 and DU145, despite both originating from the same cancer type.^291^ To fully understand disease mechanisms and MoAs for drugs, it is critical to investigate cellular activity at the single‐cell level.

Single‐cell analysis is commonly used to describe population diversity, track cell lines, classify cell types, and perform genomic analysis of rare cells.^292^ Single‐cell sequencing can effectively elucidate genomic, epigenomic, and transcriptomic heterogeneity within cell populations.^285^, ^293^, ^294^ For instance, single‐cell RNA sequencing (RNA‐seq) has demonstrated intra‐tumor heterogeneity in primary glioblastoma.^295^, ^296^ Additionally, single‐cell technologies can be integrated with proteomics, using methods like cytometry by time‐of‐flight to analyze the expression of surface and intracellular proteins.^297^, ^298^ These techniques are vital for identifying key cell subtypes relevant to drug targeting and disease progression. For example, single‐cell TargEt analysis, which combines transcriptomics and chemical proteomics, can identify cell‐specific targets and pathways. This approach has been used to uncover cell‐specific targets of aristolochic acid I in proximal tubular cells and aspirin in brain tissues.^299^ Single‐cell analysis is flourishing in various omics disciplines. However, the correlation between genotype and phenotype remains a pressing issue that requires further investigation.

**FIGURE 8:**
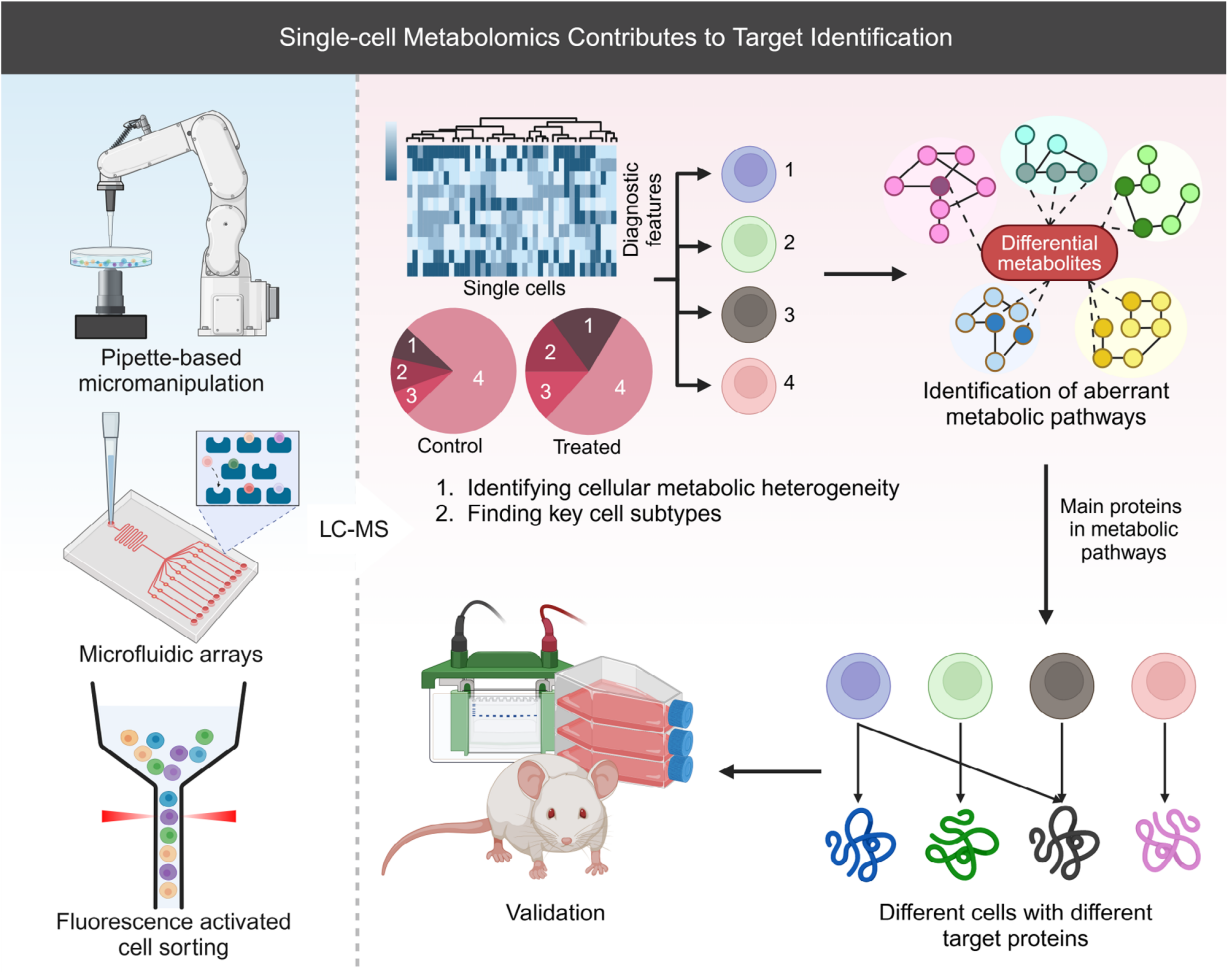
Process of target identification by single‐cell metabolomics. It can clarify the reasons for poor clinical effects of drugs or disease recurrence due to cellular heterogeneity, providing a better reference for targeted therapy (created by Biorender; https://biorender.com).

The development of single‐cell technologies has led to the emergence of single‐cell metabolomics (SCM), which examines the distinct metabolite compositions of individual cells in varying environments. SCM provides insights into the metabolic patterns of cancer cells and helps identify potential therapeutic targets (Figure 8). Studies have shown that by understanding the metabolic patterns observed in cancer cells, immune cells can be rendered more functional by adapting to the tumor microenvironment.^284^ For example, Yang et al.^300^ used single‐cell MS (SCMS) to study irinotecan (IRI)‐resistant cancer cells and discovered that combining IRI with metformin significantly inhibited fatty acid synthase, overcoming IRI resistance. They also identified distinct lipids and metabolites exclusively present in live single cells. while the upregulation of stearoyl‐coenzyme A desaturase 1 (SCD1) in IRI‐resistant cells demonstrated that inhibiting SCD1 activity increased the sensitivity of drug‐resistant cells.^301^ Furthermore, Yang et al.^302^ combined SCM and utilized a single‐probe SCMS technique to gather metabolic profiles of living cancer cells exhibiting varying levels of chemotherapy‐induced resistance. While SCM platforms have also been used to discover cell‐type‐selective microbial metabolites^303^ and reveal distinct lysosomal isoforms with unique metabolic profiles,^304^ offering new avenues for drug discovery.

Despite its potential, SCM still faces challenges, particularly in sampling strategies.^97^ Common single‐cell isolation methods include pipette‐based micromanipulation, fluorescence‐activated cell sorting (FACS), and microfluidic arrays.^305^, ^306^, ^307^, ^308^ FACS and microfluidics are preferred for preserving cell morphology and scalability,^305^, ^307^ while micromanipulation minimizes disruption to the cell environment.^307^ SCM requires higher sensitivity than conventional metabolomics due to the limited sample size of a single cell. The ability to resolve this sensitivity issue will determine the feasibility of using SCM in discovery‐based studies to gain deep biological insights.^284^, ^309^ Advances in MS, MSI, microfluidic platforms,^310^ and fluorescent biosensors have improved detection sensitivity, allowing for the simultaneous determination of hundreds of metabolites in single cells.^311^ For example, the nano‐electrospray ionization MS technique developed by Shi et al.^291^ enables simultaneous detection of nonpolar and polar metabolites at low concentrations (10 pg/mL), significantly enhancing detection coverage. Qian et al.^312^ have successfully employed a combination of flow cytometry separation and nanoparticle‐enhanced MS for high‐throughput profiling of hematopoietic stem cells, known as Hi‐scMet.

In recent years, the integration of SCM and other omics techniques has enabled the analysis of multiomics data at a finer resolution, facilitating the visualization of intricate interactions among various cellular populations.^313^ Fang et al.^314^ developed a “one‐shot” method for simultaneous proteome and metabolome analysis, uncovering the role of the NASP gene in tumor cell response to adriamycin. This technique minimizes sample loss and enhances detection sensitivity compared with traditional methods, holding significant promise for future research.^314^

However, SCM still faces limitations, such as challenges in isolating single cells without metabolite leakage and the rapid transformation of metabolites, which can lead to undetectable or fluctuating concentrations.^305^, ^307^ Additionally, the lack of standardized data‐sharing platforms hinders collaboration and further advancement.^307^ Despite these obstacles, SCM's ability to analyze cellular heterogeneity holds great promise for guiding therapeutic approaches, discovering new drug targets, and understanding disease mechanisms.

#### 4.3.3 AI‐assisted metabolomics to identify small molecule targets

**FIGURE 9:**
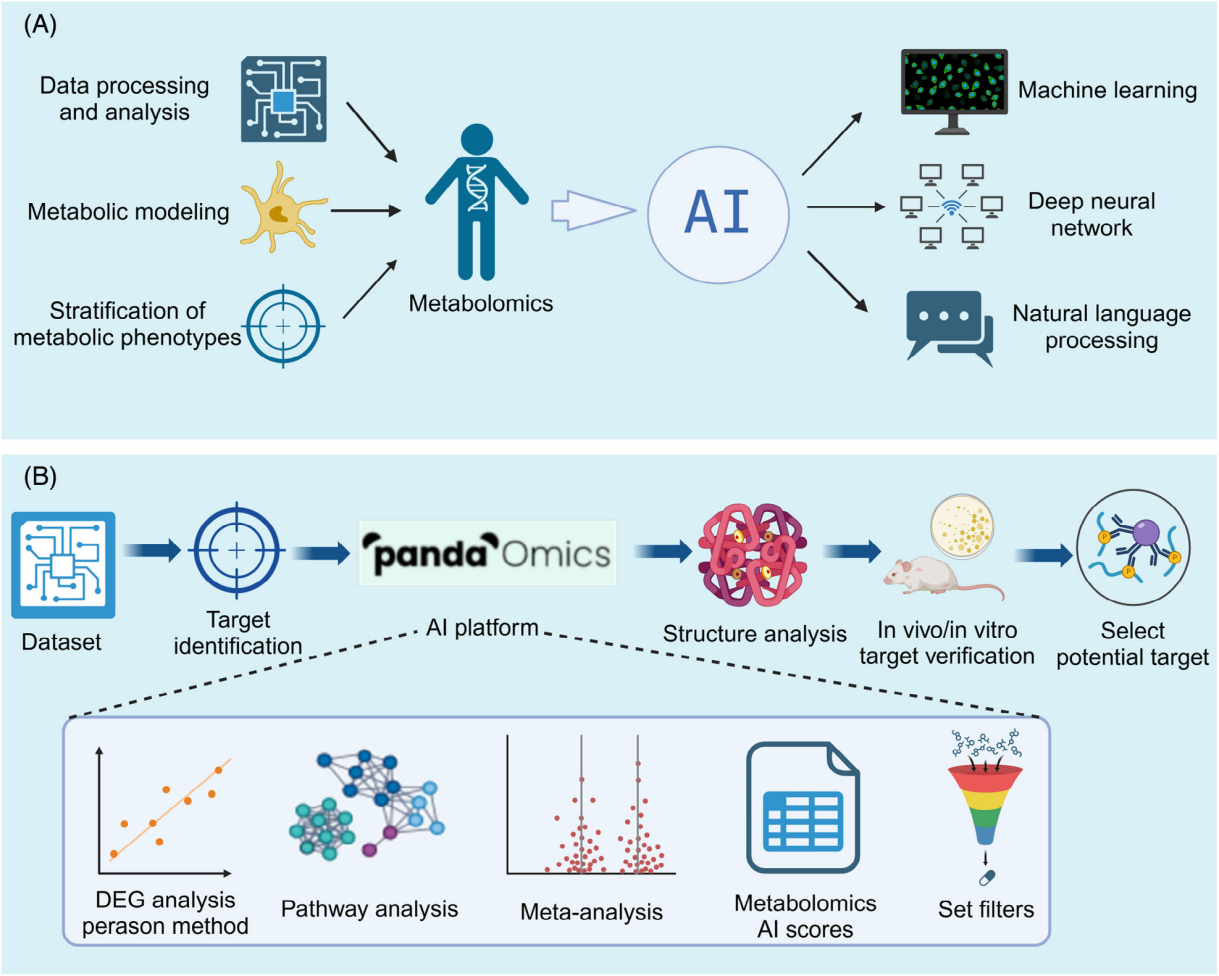
AI‐assisted metabolomics to identify small molecule targets. (A) AI integrates metabolomics data by analyzing large datasets and complex biological networks to reveal complex relationships in metabolic processes. (B) Example diagram of PandaOmics uses metabolomics to inform drug design and development for precise target identification (created by Biorender; https://biorender.com).

The vast amount of data generated in metabolomics presents a significant challenge in extracting meaningful insights. Recent advances in AI have increasingly impacted the life sciences, particularly in identifying metabolomics‐related targets.^315^ AI, through machine learning (ML) and deep learning (DL) algorithms,^316^ excels at analyzing large datasets and complex biological networks, facilitating the integration of metabolomics data and uncovering intricate metabolic relationships (Figure 9A). ML algorithms like random forest, Naive Bayes, and support vector machines (SVM) have been applied in drug development, target prediction, and disease modeling.^317^, ^318^ For instance, the Bayesian ANalysis to determine Drug Interaction Targets platform spans the drug discovery process, from target identification to drug development.^319^ Similarly, Dong et al.^320^ introduced iMSEA, an enrichment analysis strategy that enhances understanding of drug‐metabolite interactions and predicts drug side effects. ML has also proven effective in disease classification and prognosis,^321^ as demonstrated by Hu et al.^322^ in gastric cancer, offering a precision medicine tool.

ML has shown immense potential for predicting unknown metabolic pathways. Shah et al.^323^ proposed a DL‐based method for metabolic pathway prediction, utilizing annotated genomic data to train models for accurate prediction of unknown metabolic pathways, offering a novel way to unveil the intricate metabolic networks within organisms. Valentino et al.^324^ focused on the application of neural networks in metabolome pathway analysis, efficiently processing complex metabolome data and mining potential metabolic pathway information. Additionally, Hero et al.^325^ and Liu et al.^326^ have worked on improving the prediction accuracy and efficiency of metabolic pathways through the optimization of model structures and parameters. Marti et al.^326^ proposed a dynamic prediction method to analyze time‐series omics data, providing insights into the dynamic nature of metabolic processes.^327^

DL, using neural networks to extract features from large datasets, has also been employed in metabolomics research.^328^ PandaOmics, an AI‐driven drug discovery platform, combines metabolomics and multimodal DL techniques to accelerate target identification and optimize drug discovery^329^ (Figure 9B). Tools like Microsoft's BioGPT leverage large language models to mine biomedical text, linking diseases, genes, and biological processes to rapidly identify potential drug targets.^330^ To address the challenges in clinical metabolomic data analysis, such as high dimensions, small sample sizes, and class imbalances, Sha et al.^331^ proposed MetDIT, a deep convolutional neural network. MetDIT can efficiently process clinical metabolomics data, converting 1D data into 2D images for better analysis, though it highlights the need for target enrichment.^331^ Furthermore, OTTER, a novel tool, enriches and ranks targets by integrating omics and textual data, providing more accurate results for pharmacological research. OTTER provides a “keyword” that represents the pharmacological features observed in cells treated with active compounds, enabling OTTER to provide more accurate and relevant target enrichment results.^332^

AI has dramatically accelerated metabolomics research, improving data analysis accuracy while reducing costs. The application of AI in metabolomics target discovery not only significantly accelerates the research process, improves the accuracy of data analysis, but also greatly reduces the research cost. With advanced technologies such as ML and deep neural networks, the researchers are able to deeply analyze the complex relationships in metabolic processes, providing strong support for the diagnosis and treatment of diseases. However, the widespread adoption of AI in metabolomics research also faces several challenges, including ethical considerations, data privacy protection, data availability, and ensuring AI fairness and transparency.^333^, ^334^ As technologies such as interpretable AI evolve and more metabolomics data becomes available, AI is expected to revolutionize drug discovery and target identification.

## 5 PERSPECTIVES

The interplay between genetic information and metabolic disorders is fundamental to the pathogenesis of many diseases. The abnormalities in upstream genetic processes can lead to disruptions in metabolic pathways, which accordingly mediate the onset and progression of various diseases. Therefore, focusing on aberrant metabolic pathways and elucidating the intricate mechanisms of metabolic signal transduction, we can not only uncover novel therapeutic avenues by identifying potential targets, but also inspire innovative strategies that target bifunctional metabolic enzymes or the intricate transcriptional axes of complex metabolic genes.^335^ One compelling reason why metabolomics is emerging as a crucial tool for identifying therapeutic targets lies in its ability to pinpoint the key metabolic pathways and enzymes by detecting variations in metabolic profiles.

Acknowledging the intricacies of metabolic pathways and the multitargeting trait of drugs, metabolomics has evolved with novel analytical methods, including high‐throughput metabolomics for comprehensive profiling, dose–response metabolomics exploring concentration effects, and SIRM for precise metabolic traci. The rapid analysis capabilities and the ability to handle large sample sizes of high‐throughput metabolomics underpin robust genome‐wide predictions of drug–target interactions. Dose–response metabolomics establishes a link between drug concentration and sample response, facilitating the evaluation of various drugs’ impacts on proteins, offering valuable insights into the underlying biochemical mechanisms and unintended off‐target effects. While SIRM bridges the gap in traditional metabolomics by providing insight into specific metabolic pathways via stable isotope labeling. This advancement enables a dynamic characterization of metabolism and MFA within simulated metabolic networks, empowering metabolic flux techniques to elucidate the interplay between metabolic and functional states in vivo. Consequently, it facilitates the precise identification of promising therapeutic targets.

Furthermore, owing to cellular heterogeneity, cutting‐edge technologies such as single‐cell analysis has been integrated in metabolomics, providing a more direct and dynamic depiction of cellular phenotypes. The continuous advancements in microfluidics, micromanipulation, image analysis, and automation facilitate the high‐throughput isolation of individual cells without damaging cellular metabolism, thereby allowing for the most accurate description of cellular reaction networks. Despite the current scarcity of mature processes and methodologies for single‐cell metabolomics aimed at pinpointing potential therapeutic targets, the enhanced resolution and broader perspective have revolutionized the insights into disease mechanisms and MoAs. With the advances in computational approaches and network models, the integration of AI streamlines the analysis vast data sets, empowering researchers to extract meaningful insights.^336^ Furthermore, in recent years, other cutting‐edge technologies such as MSI have been actively employed in target identification, progressively solidifying metabolomics as a crucial tool for drug target identification.

In addition to targeting proteins and enzymes for disease treatment, a diverse range of alternative MoAs are employed. These encompass disrupting membrane structures, scavenging ROS,^337^ employing hypoxic‐targeted therapies,^338^ and utilizing prodrugs. However, it is undeniable that these methods, whether directly or indirectly, induce alterations in cellular metabolism. While metabolomics may not precisely pinpoint the core mechanisms or key sites of drug action, it provides valuable insights by exploring the complex metabolic network. Subsequently, these findings can be corroborated through complementary biological experiments.

Indeed, metabolomics still faces several enduring challenges across its diverse approaches. First, common challenges include metabolite annotation, inadequate coverage, and degradation of active metabolites, which exist across in various metabolomics‐based extension methods. Although optimizing extraction protocols and incorporating AI have improved the current situation, further advancements are still imperative. Second, multiomics strategies also pose unique challenges, including sample collection and processing, integration and normalization of disparate datasets, as well as conducting comprehensive analysis across different omics layers. Additionally, high‐throughput metabolomics frequently necessitates compromising between metabolite coverage, isomer separation, and efficiency. Balancing these trade‐offs while enhancing overall performance remains a formidable challenge that necessitates continuous refinement. Despite these challenges, metabolomics has undeniably emerged as a vital tool in unraveling disease mechanisms and advancing drug discovery.

## AUTHOR CONTRIBUTIONS

Tengfei Xu conceived the article. Shanshan Pan and Luan Yin mainly wrote the manuscript. Jie Liu, Jie Tong, ZichuanWang, and Jiahui Zhao edited and proofread the manuscript. Xuesong Liu, Yong Chen, Jing Miao, and Yuan Zhou revised the article and provided important feedback. Tengfei Xu and Su Zeng assisted in the writing and reviewing the manuscript. All authors have read, discussed and approved the article.

## CONFLICT OF INTEREST STATEMENT

The authors declare no conflict of interest.

## ETHICS STATEMENT

Not applicable.

## Data Availability

Not applicable.
